# Evaluation of the *FAS* and *FASL* Gene changes in women with premature ovarian failure: A case-control study

**DOI:** 10.18502/ijrm.v20i12.12561

**Published:** 2023-01-09

**Authors:** Zhima Akhavansales, Alimohammad Mosadeghmehrjardi, Hamid Reza Ashrafzadeh, Shadnaz Fakhteh Yavari, Mohammad Taher Tahoori, Morteza Bitaraf Sani, Mahnaz Mohammadi, Fateme Montazeri, Nasrin Ghasemi

**Affiliations:** ^1^Department of Immunology, Faculty of Medicine, International Campus, Shahid Sadoughi University of Medical Sciences, Yazd, Iran.; ^2^Department of Traditional Pharmacy, Faculty of Traditional Medicine, Tehran University of Medical Sciences, Tehran, Iran.; ^3^Abortion Research Center, Yazd Reproductive Sciences Institute, Shahid Sadoughi University of Medical Sciences, Yazd, Iran.; ^4^Department of Tissue Engineering and Applied Cell Science, School of Advanced Technologies in Medicine, Shahid Beheshti University of Medical Science, Tehran, Iran.; ^5^Department of Immunology, Faculty of Medicine, Shahid Sadoughi University of Medical Sciences, Yazd, Iran.; ^6^Animal Science Research Department, Yazd Agricultural and Natural Resources Research and Education Center, Agricultural Research, Education & Extension Organization (AREEO), Yazd, Iran.

**Keywords:** FAS, FASL, Polymorphism, Premature ovarian failure.

## Abstract

**Background:**

Premature ovarian failure (POF), is menopause occurring before the age of 40, affecting 1-3% of women worldwide. The risk of POF increases with altered immunological parameters such as *FAS *and *FASL* genes, which play a fundamental role in embryogenesis and cellular homeostasis.

**Objective:**

The study aimed to investigate the potential role of *FAS* and *FASL *genes in POF pathogenesis.

**Materials and Methods:**

In this case-control study, the polymorphisms of *FAS*-670A/G and *FASL*IVS2nt_124A/G apoptotic genes were analyzed in 51 Iranian women suffering from POF, and 61 healthy controls. Isolation of DNA was done using the salting-out method, and genotypic analysis was performed for all the subjects using the polymerase chain reaction-restriction fragment length polymorphism method.

**Results:**

Our results revealed that homozygous *FAS*-670A/A and G/G, and heterozygous *FAS*-670A/G are not significantly different between cases and controls (p = 0.99). Also, in different genotyping models of *FAS*IVS2nt_124, polymorphisms were not related to POF risk (p = 0.23).

**Conclusion:**

There is no statistical association between these polymorphisms and POF risk in women referred to genetic counseling clinics.

## 1. Introduction

Infertility is a multifactorial disease that ranges from hormonal and genetic disorders to immunological changes. A gradual decrease in ovarian function is observed as menopause nears, leading to estrogen deficiency and a decrease in fertility. This process is associated with higher gonadotropin levels (1-3). Premature ovarian failure (POF), is a menopause that occurs before the age of 40, affecting 1-3% of women worldwide (4, 5). The development and maturation of ovulation depend on molecular signaling pathways responding to androgens (6, 7).

Estrogen is one of the 2 steroid sex hormones secreted by ovaries. Estrogen also has an important role in fetal development, the presence of secondary sexual features, the reproductive cycle, and the continuation of pregnancy. In addition, estrogen regulates the growth and differentiation of endometrial cells (8). In the reproductive cycle, implantation involves a series of events, including apoptosis in endometrial cells (9, 10). Evidence suggests that apoptosis helps maintain cellular homeostasis by removing senescent cells from the functional layer of the uterine endometrium (11).

*FASL* acts as a mediator of apoptosis between cell differentiation and embryonic development. It has been reported that polymorphisms in *FAS* and *FASL* have clinical worth in hormone-sensitive cancers such as ovarian and breast cancer. Estrogen, one of the most important sex hormones, increases the expression of the *FASL* protein (12). Genetic tests also show increased *FASL *mRNA expression by estradiol and progesterone. It was shown that increased *FASL *expression may mediate apoptosis in endometrial cells (9). Therefore, it can play an important role in trophoblast invasion and consequent implantation.* FAS* and *FASL *genes have many polymorphisms even in the gene promoter, which could be important in regulating the cell death signal (13-15).

The *FASL* gene has 4 exons. *FASL*INV2nt_124A/G rs5030722 is one of the most important polymorphisms reported in this gene, located within the intron. Considering the effect of estrogen on *FAS* and *FASL* regulation, we investigated the effect of standard *FAS* and *FASL* polymorphisms with POF.

## 2. Materials and Methods

### Study subjects 

Among 112 women who were admitted to the Recurrent Abortion Clinic of Yazd Reproductive Sciences Institute, Yazd, Iran 51 women met the diagnostic criteria of POF and 61 healthy women were considered as case control groups, respectively.

### Genotype analysis

Genomic DNA was extracted from 5 ml of whole blood by the salting out method (16). DNA was amplified through polymerase chain reaction with designed primers of *FAS *and *FASL* genes (Table I). The solution for polymerase chain reaction was made by 30 ng of genomic DNA, 10 p moles from each primer (Macrogen Inc., Korea), 10 μl of Master Mix RED (AMPLIQON, Denmark), plus distilled water up to 20 μl as the final volume. The reaction starts by denaturation for 5 min at 95 C, followed by 2 steps involving 10 cycles as the first step and 25 cycles as the second step. The first step contains 30 sec of denaturation at 95 C, 50 sec of annealing at 62 C, and 40 sec of extension at 72 C, and the second step contains 20 sec of denaturation at 95 C, 50 sec of annealing at 58 C, and 40 sec of extension at 72 C. The final extension was done within 3 min at 72 C. The products included 193 bp for *FAS*-670A/G and 230 bp for *FASL*_124G/A. They were restricted by MvaI (Fermentase, Germany) and FokI (BIOLAB, Germany) enzymes, respectively at 37 C overnight. The products were stained by safe staining and separated on 2.5% agarose gel electrophoresis, resulting in 136 and 57 bp fragments in the* -*670G allele and 180 and 50 bp fragments in the *FASL*IVS2nt*_*124G allele (17, 18).

**Table 1:** The primers used for the ampliﬁcation of target genes


**SNP**	**Forward**	**Reverse**
*FAS-670A/G*	5'-ATAGCTGGGGCTATGCGATT-3'	5'-CATTTGACTGGGCTGTCCAT-3'
*FASL_124G/A*	5'-GCAGTTCAGACCTACATGATTAGGAT-3'	5'-CCAGATACAGACCTGTTAAATGGGC-3'
SNP: Single nucleotide polymorphism		

### Ethical considerations

This study was approved by the ethics committee of Yazd Reproductive Science Institute, Yazd, Iran (Code: IR.SSU.RSI.REC.1396.27). The women were informed of the research goal and signed the consent form. Women with unplanned pregnancies and those who received assisted reproductive technologies were omitted from our study.

### Statistical analysis

The frequency of the alleles, genotypes and haplotype in cases and controls were compared by Chi-square test (p-value < 0.05 was significant). Fisher's exact test, Two-sided p-values, and odds ratio (OR) (with 95% confidence interval) were calculated by SPSS statistical software (Version 20). Genetic models (i.e., dominant, codominant, recessive, and overdominant) were analyzed using R software (the SNPassoc package) to assess the relationship between alleles. The PLINK software was employed for haplotype blocks.

## 3. Results

The genotype distribution of the 2 polymorphisms from both cases and the controls were in line with Hardy-Weinberg equilibrium (p > 0.05).

### Allele frequencies

The allelic odds ratio of *FAS*-670A/G and *FASL*_124G/A were 1.004 and 1.145, respectively. No significant difference was observed between the allele frequencies of SNPs in this research (Table II). Allele frequencies within cases and controls are shown in table II. Also, odds ratio statistics with p-value for any allele were presented. Data were presented in percentages.

### Genotype frequencies

Regarding the *FASL*IVS2nt_124, the frequency of AG genotype was higher in the controls (26.7%) than in cases (17.6%). However, the difference was not statistically significant (p = 0.23). Most POF women had AA genotype relative to *FASL*IVS2nt_124AG and GG genotypes (72.5%, 17.6% and 9.8%, respectively). Under the codominant model, the frequency of the *FAS-*670AG genotype was not significantly different between cases (47.1%) and controls (46.7%) (p = 0.99). Also, the AG genotype was the most common genotype in POF women (AG = 47.1%, GG = 31.4%, and AA = 21.6%) (Table III). No significant difference was observed in genotype frequencies of *FASL*IVS2nt*_*124 A/G and *FAS-*670 A/G variants between women with POF and healthy controls.

### Haplotype frequency 

Haplotype analysis was done for these SNPs to investigate the association between a likely combination of *FASL*IVS2nt_124A/G and *FAS* *-*670A/G polymorphisms with POF. Most women with POF had GA and AA haplotypes (0.45% and 0.35%, respectively) (Table IV). There was no significant difference in the frequency of haplotypes between cases and controls.

**Table 2:** Frequency distribution of *FAS* alleles in 2 groups and their relationship with POF risk


**Model**	**Allele**	**Cases**	**Controls**	**OR (95% CI)**	**P-value**
	A	0.451	0.450	
*FAS-670A/G*	G	0.549	0.550	1.004 (0.47-2.12)	0.988
		A	0.186			0.166	
*FAS-124G/A*	G	0.814	0.834	1.145 (0.60-2.09)	0.702
	Fisher's exact test was used to survey significant of odds ratios					

**Table 3:** Genotype frequency distribution of *FAS* SNPs between cases and controls


**Model**		**Genotype**	**Cases (%)**	**Controls (%)**	**OR (95% CI)**	**P-value**
*FAS-670A/G*						
	**Codominant**	G/G	0.314	0.317	1.00	0.9997
	**Heterozygote**	A/G	0.471	0.467	1.00 (0.42-2.39)	
	**Homozygote**	A/A	0.216	0.217	1.01 (0.35-2.90)	
	G/G	0.314	0.317	1.00	
	**Dominant**	A/G-A/A	0.686	0.683	1.00 (0.44-2.26)	0.9942
		G/G-A/G	0.784	0.783	1.00		
	**Recessive**	A/A	0.216	0.217	1.01 (0.40-2.53)	0.9793
		G/G-A/A	0.529	0.533	1.00		
	**Overdominant**	A/G	0.471	0.467	0.99 (0.47-2.12)	0.9883
*FASL_124 A/G*						
	**Codominant**	A/A	0.725	0.700	1.00	0.2346
	**Heterozygote**	A/G	0.176	0.267	0.64 (0.25-1.6)	
	**Homozygote**	G/G	9.8	3.3	2.84 (0.52-15.57)	
	A/A	0.725	0.700	1.00	
	**Dominant**	A/G-G/G	27.5	30.0	0.88 (0.39-2.02)	0.7661
		A/A-A/G	0.902	0.967	1.00		
	**Recessive**	G/G	0.098	0.033	3.16 (0.58-17.08)	0.1587
		A/A-G/G	0.824	0.733	1.00		
	**Overdominant**	A/G	0.176	0.267	0.59 (0.23-1.48)	0.2535
P-values < 0.05 were significant						

**Table 4:** *FAS* and *FASL* haplotypes of POF women and controls


**Haplotype**	**Cases (%)**	**Controls (%)**	**P-value**
**AG**	0.091	0.060	0.383
**GG**	0.094	0.105	0.780
**AA**	0.359	0.389	0.646
**GA**	0.454	0.444	0.877
Haplotype analyses was carried out using Chi-square test and using The PLINK software. P-values < 0.05 were significant			

## 4. Discussion

Apoptosis is vital for the formation of tissue structure during embryogenesis and cellular homeostasis, and it serves as a defense mechanism in facing pathogens (19, 20). Apoptosis is simultaneous with the implantation window (7). The expression of *FASL* gene is a important source of apoptosis, which changes in the ovary in female reproductive cycles. The levele of the *FASL* protein is corresponding to the estrogen-receptor beta-expression. Estrogen up-regulates the expression of *FASL *and higher mRNA finds in normal ovarian epithelial cells (21).

It has been suggested that regulation of *FASL* expression in human endometrium is dependent on steroid sex hormones. In immunohistochemistry tests, a gradual increase in the immune reaction of *FASL* has been observed in both stromal and glandular cells, and the strong expression of *FASL* has been detected in late reproductive and secretory phases (9). In 2006, Slot and colleagues concluded in their experiments that the expression of *FASL* in normal ovaries was hormone-sensitive, and could play a key role in the physiology of normal ovarian tissue. They also found that estrogen is an important sex hormone, increasing the expression of *FASL* protein (21). Genetic tests have indicated an increase in the expression of *FASL* mRNA by estradiol and progesterone. It was revealed that higher *FASL* expression may mediate apoptosis in endometrial cells and can thus play an important role in trophoblast invasion and consequent implantation (9). It is believed that estrogen plays a vital role in oocyte maturation and fertilization (6). Disruption or polymorphism of apoptosis-related genes can impair the relevant immune responses (8).

Transcriptional activity of these genes change by Single-nucleotide polymorphisms in the promoter region. The -670A/G polymorphism of the *FAS* gene lies in the transcription binding site for the 2 main transcription factors of Sp1 and STAT1. Besides, *FASL*INV2nt_124A/G, rs5030772 in intron 2 of this gene have a important influence on the control of the expression of *FASL* (22). Considering the above statements, we evaluated the role of *FAS* and *FASL* genes polymorphisms in POF pathogenesis. Nevertheless, *FASL* expression in the normal ovary is sensitive to hormones and it could have a major role in the physiology of normal ovarian tissue (21). Based on our data, neither *FAS* nor *FASL* polymorphisms seem to be susceptibility factors for POF in our population.

## 5. Conclusion

This study showed that the gene variants under research were not involved in the pathogenesis of POF in the Iranian population. However, this does not completely exclude such genes as potential candidates for the occurrence of this disease. A comprehensive genetic analysis of the genes implicated in the intricate apoptosis regulation system could result in the identification of susceptibility factors for the disease and a better understanding of its etiology.

##  Conflict of Interest

The authors declare that there is no conflict of interest.
